# Genomic instability of TnSMU2 contributes to *Streptococcus mutans* biofilm development and competence in a *cidB* mutant

**DOI:** 10.1002/mbo3.934

**Published:** 2019-10-09

**Authors:** Matthew E. Turner, Khanh Huynh, O’neshia V. Carney, Dennis Gross, Ronan K. Carroll, Sang‐Joon Ahn, Kelly C. Rice

**Affiliations:** ^1^ Department of Microbiology and Cell Science Institute of Food and Agricultural Sciences University of Florida Gainesville FL USA; ^2^ Department of Biological Sciences Ohio University Athens OH USA; ^3^ Department of Oral Biology College of Dentistry University of Florida Gainesville FL USA

**Keywords:** Cid/Lrg system, competence, genomic island, oxidative stress, *Streptococcus mutans*, TnSMU2

## Abstract

*Streptococcus mutans* is a key pathogenic bacterium in the oral cavity and a primary contributor to dental caries. The *S. mutans* Cid/Lrg system likely contributes to tolerating stresses encountered in this environment as *cid* and/or *lrg* mutants exhibit altered oxidative stress sensitivity, genetic competence, and biofilm phenotypes. It was recently noted that the *cidB* mutant had two stable colony morphologies: a “rough” phenotype (similar to wild type) and a “smooth” phenotype. In our previously published work, the *cidB* rough mutant exhibited increased sensitivity to oxidative stress, and RNAseq identified widespread transcriptomic changes in central carbon metabolism and oxidative stress response genes. In this current report, we conducted Illumina‐based genome resequencing of wild type, *cidB* rough, and *cidB* smooth mutants and compared their resistance to oxidative and acid stress, biofilm formation, and competence phenotypes. Both *cidB* mutants exhibited comparable aerobic growth inhibition on agar plates, during planktonic growth, and in the presence of 1 mM hydrogen peroxide. The *cidB* smooth mutant displayed a significant competence defect in BHI, which was rescuable by synthetic CSP. Both *cidB* mutants also displayed reduced XIP‐mediated competence, although this reduction was more pronounced in the *cidB* smooth mutant. Anaerobic biofilms of the *cidB* smooth mutant displayed increased propidium iodide staining, but corresponding biofilm CFU data suggest this phenotype is due to cell damage and not increased cell death. The *cidB* rough anaerobic biofilms showed altered structure relative to wild type (reduced biomass and average thickness) which correlated with decreased CFU counts. Sequencing data revealed that the *cidB* smooth mutant has a unique “loss of read coverage” of ~78 kb of DNA, corresponding to the genomic island TnSMU2 and genes flanking its 3′ end. It is therefore likely that the unique biofilm and competence phenotypes of the *cidB* smooth mutant are related to its genomic changes in this region.

## Graphical abstract

*Streptococcus mutans* employs numerous physiological strategies to compete and persist within the oral cavity. In this report, we identify and characterize two stable *cidB* mutant colony variants with respect to their oxidative stress resistance, biofilm formation, and competence phenotypes. These studies further define a role for *cidB* in each of these physiological functions, and genome resequencing of each *cidB* mutant variant reinforces a previously observed connection between the *cid* operon and genomic island TnSMU2.

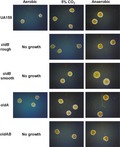

## 1 INTRODUCTION

Although dental caries is considered a microbial shift disease within the human oral cavity (Marsh, 2006; Mira, Simon‐Soro, & Curtis, 2017; Sanz et al., 2017), *Streptococcus mutans* is highly associated with caries formation (Garcia et al., 2017; Loesche, 1986). *S. mutans* utilizes a variety of carbohydrates to power a fermentation‐based metabolism featuring adaptive pyruvate usage based on the growth environment (Abbe, Takahashi, & Yamada, 1982; Yamada & Carlsson, 1975; Yamada, Takahashi‐Abbe, & Abbe, 1985). Acidic end products from this metabolism, including lactic acid (Hojo, Komatsu, Okuda, Takahashi, & Yamada, 1994), build up in complex biofilms attached to the tooth surface, leading to the dissolution of enamel, and the formation of dental caries. Biofilm formation is a major virulence factor of *S. mutans*. Extracellular polysaccharide (EPS) synthesized by glucosyltransferases (GTFs) help the organism attach to tooth surfaces (Tsumori & Kuramitsu, 1997) and entomb the bacterium in a complex matrix that can protect it from antibiotics and fluid shearing (Fernández, Aspiras, Dodds, González‐Cabezas, & Rickard, 2017; Shen, Stojicic, & Haapasalo, 2011). Biofilms also form an intricate microenvironment limiting oxygen exposure (Wessel et al., 2014), sequestering nutrients (Hwang et al., 2016), and establishing pH gradients (Xiao et al., 2012). Extracellular DNA (eDNA) is also a major component of oral biofilms. Reports have shown eDNA to be distributed widely in *Enterococcus faecalis* biofilms, as well as biofilms generated from clinically collected human saliva samples (Rostami et al., 2017). The presence of eDNA is also important for *S. mutans* biofilm formation and regulated cell death (Perry, Cvitkovitch, & Lévesque, 2009).

Oral streptococci such as *S. mutans* can also utilize eDNA for genetic exchange via natural competence. In *S. mutans*, two quorum‐sensing systems have been characterized in regulation of competence. In peptide‐rich growth media, the competence‐stimulating peptide (CSP, encoded by *comC*)‐based system is used. CSP is a 21AA peptide which is released into the extracellular environment via ComAB. The peptide is then processed by the protease SepM (Hossain & Biswas, 2012) into a final 18AA form which can be recognized by the ComDE two‐component system. ComD is a histidine kinase which phosphorylates ComE, a response regulator, which then can activate bacteriocin genes, such as *nlmC,* and provide negative regulatory feedback for *comC* (Kreth et al., 2007). Phosphorylated ComE indirectly leads to *comX* activation, the alternative sigma factor involved in the regulation of competence genes (Li et al., 2002), yet the process by which this occurs remains unclear. Alternative to CSP, a 7AA peptide identified as *comX*‐inducing peptide (XIP) is processed from ComS, which can directly activate *comX* expression through the regulator ComR. Exported XIP is imported via the Opp permease and forms a complex with ComR, which then binds upstream of the gene encoding the XIP peptide, *comS*, and *comX* (Mashburn‐Warren, Morrison, & Federle, 2010). Opp‐mediated uptake is blocked by peptide‐rich media, but ComS has also been demonstrated to participate in competence signaling without leaving the cell (Underhill et al., 2018). While both CSP and XIP induce competence in alternative ways, addition of synthetic forms of either peptide has been shown to stimulate a *comX* response (Son, Ahn, Guo, Burne, & Hagen, 2012).

The *S. mutans cidAB* transcriptional unit encodes two proteins (CidA and CidB) which have been shown to affect a multitude of virulence traits including biofilm development and oxidative stress response (Ahn, Rice, Oleas, Bayles, & Burne, 2010). Expression of *cidAB* has also been shown to be regulated by catabolite control protein A (CcpA; Kim, Waters, Turner, Rice, & Ahn, 2019). While the close homologs of CidA/B, LrgA/B, have recently been characterized in *Bacillus subtilis* as a transporter of pyruvate (Charbonnier et al., 2017; Esker, Kovács, & Kuipers, 2017), the exact function of the Cid/Lrg proteins in *S. mutans* remains unknown. Expression data have shown that *cidAB* RNA levels are upregulated during anaerobic growth (Ahn, Wen, & Burne, 2007) and by growth in the presence of excess glucose (Ahn et al., 2010). Transcriptomic data collected under anaerobic conditions also suggest an interesting effect on the expression of genomic islands (GIs) TnSMU1 and TnSMU2 (Ajdić et al., 2002) of *S. mutans* in a *cidB* mutant (Ahn & Rice, 2016). Many of the genes encoded within these regions were found to be significantly upregulated in a *cidB* mutant as compared to wild type under the same conditions, possibly indicating a link between Cid and these mobile genetic elements (MGEs).

Genomic islands are a major class of MGE commonly acquired via horizontal gene transfer events. There are two identified GIs within the *S. mutans* UA159 genome designated TnSMU1 and TnSMU2 (Ajdić et al., 2002). TnSMU2 is large, consisting of over 50 kb, and is flanked by transposase remnants. It also has a distinct shift in G + C nucleotide content (~28%) relative to the rest of the *S. mutans* genome (37.5%; Waterhouse & Russell, 2006). The primary coding regions within this island have been characterized as a cluster containing nonribosomal peptide synthases, polyketide synthases, and accessory proteins involved in pigment synthesis as well as oxidative stress tolerance and biofilm formation (Wu et al., 2010). The region also contains a TetR‐like regulator, SMU.1349, which can act as both as an activator for TnSMU2 genes and as a repressor of its own expression (Chattoraj, Mohapatra, & Rao, 2011). It is also notable however that TnSMU2 is not present in all *S. mutans* strains, with several studies reporting a range of clinical isolates failing to generate PCR products with primers targeted to this GI (Lapirattanakul et al., 2008; Wu et al., 2010).

During routine culturing of the *S. mutans cidB* mutant from different laboratory frozen glycerol stock cultures, we recently noticed the presence of two stable phenotypes based on colony morphology: a “rough” variant and a “smooth” variant. While the rough variant had been utilized for previous studies characterizing the physiological role of *cidB* (Ahn & Rice, 2016; Ahn et al., 2010), discovery of the smooth variant was novel. Hi‐depth genome resequencing revealed this morphological difference may stem from the loss of TnSMU2, as well as an additional ~20 kb on the island's 3′ end. In this report, we explore the physiological differences between the *cidB* rough and *cidB* smooth mutants in relation to the presence or loss of the TnSMU2 region, respectively. Critical *S. mutans* physiological functions such as genetic competence, oxidative and acid stress resistance, and biofilm formation were also compared between UA159 and both *cidB* mutant variants. As a whole, this work addresses the physiological role(s) that *cidB* and TnSMU2 play in *S. mutans* UA159, and reinforces a previously established regulatory connection (Ahn & Rice, 2016) between these two loci.

## 2 MATERIALS AND METHODS

### 2.1 Bacterial strains and growth conditions

For each experiment below, *S. mutans* UA159 and/or its isogenic *cidA* (∆*cidA*::Km^r^, nonpolar (NP)), *cidB* rough and smooth (*∆cidB*:: NPKm^r^), and *cidAB* (∆*cidA*:: ΩKm^r^) mutants created in (Ahn et al., 2010) were streaked from frozen glycerol stocks on Brain Heart Infusion (BHI, Oxoid, UK) containing 1 mg/ml kanamycin for the *cid* mutants. A *S. gordonii* DL‐1 pyruvate oxidase (*spxB*) mutant (Huang et al., 2016) was generously provided by the laboratory of Dr. Robert Burne (University of Florida). *S. gordonii* DL‐1 and/or its isogenic *spxB* mutant were cultured on BHI (+1 mg/ml kanamycin for the *spxB* mutant). Unless otherwise stated, all agar plates, biofilms, and planktonic *S. mutans* and *S. gordonii* cultures were grown at 37°C in a 5% CO_2_ incubator, in either BHI, semidefined biofilm medium (BM; Loo, Corliss, & Ganeshkumar, 2000) containing 20 mM sucrose, or FMC media (Terleckyj, Willett, & Shockman, 1975). *E. coli* JM110 harboring the plasmid pOri23 (Que, Haefliger, Francioli, & Moreillon, 2000) was grown in aerobic conditions (37°C, 250 RPM) in Luria‐Bertani (LB Lennox, BD) broth with erythromycin (300 µg/ml). All glycerol stock cultures were maintained at −80°C and were prepared by mixing equal volume of overnight with sterile 80% (vol/vol) glycerol in cryogenic tubes.

### 2.2 Colony morphology comparisons

To compare general trends in colony morphology between *S. mutans* UA159 and the *cidA, cidB,* and *cidAB* mutants, cells were grown 16–18 hr and then serially diluted in BHI. An equal volume (100 µl) of each 10^–7^ dilution was spread‐plated onto BHI agar plates and incubated for 48 hr at 37°C in either a regular plate incubator (atmospheric conditions), an incubator supplemented with 5% CO_2_, or in an anaerobic environment (Pouch‐Anaero anaerobic Gas Generating System, Mitsubishi Gas Chemical Company, Japan). Representative colony images from *n* = 3 independent experiments were then captured using a Zeiss Stemi 305 Microscope and corresponding Labscope software (Zeiss, Germany). All images were taken at 10× magnification using darkfield settings.

### 2.3 Genomic DNA isolation, Illumina Resequencing, and SNP analysis

To analyze and identify single nucleotide polymorphisms (SNPs) and other sequence changes, genomic DNA (gDNA) was isolated from *S. mutans* UA159, *cidB* rough, and *cidB* smooth strains. Cells were grown to late exponential phase (Optical density at 600 nm (OD_600_) between 0.5–0.8) in BHI, at which time cell pellets were harvested from 7 ml of culture by centrifugation (3,900 × *g*, 10 min). DNA extractions were performed using the Promega Wizard Genomic DNA Purification Kit (Wisconsin, USA) with the following modifications: Following cell pellet resuspension in 50 mM ethylenediaminetetraacetic (EDTA) acid, cells were lysed with 100 µl of 10 mg/ml lysozyme and 20 units mutanolysin (Sigma‐Aldrich) at 37°C, 50 RPM, for 60 min. Total gDNA was precipitated in isopropanol for 1 hr, at room temperature, on a mixing platform, and gDNA pellets were washed once with room temperature 70% ethanol and air‐dried before being solubilized per kit instructions. The gDNA concentration, A_260_/A_280_, and A_260_/A_230_ ratios were quantified using a Nanodrop (Thermo Fisher) to ensure gDNA quality: DNA concentration above 100 ng/µl, A_260_/A_280_ between 1.8 and 2.0, and A_260_/A_230_ between 2.0 and 2.2. Samples were then submitted to the University of Florida Interdisciplinary Center for Biotechnology Research (ICBR) for library construction using the NEB Ultra II PCR‐based library construction protocol. Qubit and TapeStation were performed on the final libraries to calculate nM quantity prior to pooling libraries using equimolar amounts. Genome resequencing was performed using the Illumina MiSeq 2x300 platform with Illumina v3 sequencing reagents. Sequence analysis was then performed using CLC Genomics Workbench (Qiagen) using basic variant detection with a 90% minimum frequency cutoff, as described in (Mashruwala et al., 2016).

### 2.4 Real‐time PCR (qPCR)

Relative copy number of genes located within and outside of the low‐coverage TnSMU2 region of the *cidB* smooth mutant were verified using quantitative real‐time polymerase chain reaction (qPCR). Total gDNA was isolated from wild‐type, *cidB* rough, and *cidB* smooth strains as described above and was used as template with the primer sets indicated in Table 1. qPCRs were performed using iQ SYBR Green Supermix (Bio‐Rad, California, USA), 30 ng gDNA per reaction, and with primers at 250 nM per reaction on a Bio‐Rad CFX Connect Real‐Time System with the following conditions: 95°C for 3 min and 34 cycles of 95°C for 15 s followed by 55°C for 30 s. Relative copy number was then compared using the reported C_T_ value for each reaction.

**Table 1:** qPCR primers used in this study

Primer name	Sequence
*bacA2‐F*	5′‐ACAAGTGGGCGATGTAGTTG‐3′
*bacA2‐R*	5′‐TCAATTGGCGTTCCCGAATC‐3′
*comE‐F*	5′‐CAGTATCAGGTATCTGCTTTGGA‐3′
*comE‐R*	5′‐TGACCATTCTTCTGGCTGTT‐3′
*gbpC‐F*	5′‐TGAACCAACGCCAGAAAAGC‐3′
*gbpC‐R*	5′‐CACGCTCTCTAACACGCATTTC‐3′
*pdhB‐F*	5′‐ACATGTCAGCTTCTGTTGGG‐3′
*pdhB‐R*	5′‐AACGCATTTTCGACCCTTGG‐3′

### 2.5 Aerobic growth assay

Growth of *S. mutans* UA159 and isogenic *cid* mutants in aerobic conditions was assayed using a Bioscreen C automated growth system (Growth Curves USA). *S. mutans* cells were grown 16–18 hr in a chemically defined media (FMC; Terleckyj et al., 1975) and then diluted to an OD_600_ of 0.02 per milliliter in fresh FMC. A honeycomb well plate (Growth Curves USA) was then inoculated at a 1:4 well to volume ratio, and cell optical density was recorded over 24 hr with constant shaking at 37°C.

### 2.6 Acid tolerance assay

Each strain's ability to withstand acid stress was assayed by measuring cell viability over time after exposure to low pH conditions. Cells were first grown 16–18 hr in BHI and then diluted to an OD_600_ of 0.02 in 5 ml of fresh BHI. Cultures were then grown for 4 hr before cells were harvested via centrifugation. Supernatant was then removed, and cell pellets were resuspended in 5 ml of 0.1 M glycine buffer at a pH of either 3.5 or 7, and incubated at 37°C, 5% CO_2_. Samples were then removed at 0, 20, 40, 60, and 90 min incubation and serially diluted before being plated on BHI agar. Total colony‐forming units (CFUs) were then enumerated after 48 hr growth at 37°C in a 5% CO_2_ incubator.

### 2.7 Dot drop competition assays

The ability of wild‐type and *cid* mutant strains to compete against *S. gordonii* DL‐1 and its isogenic *spxB* mutant was determined by a dot drop competition assay as described previously (Kreth, Merritt, Shi, & Qi, 2005). In brief, *S. gordonii* or *spxB* mutant cells were grown overnight for 16–18 hr in 0.5x BHI media and then diluted to an OD_600_ of 0.5 in fresh 0.5× BHI media. About 10 µl was then dropped onto 0.5× BHI (Difco, BD) agar, and the plate was incubated overnight at 37°C in 5% CO_2_. The following day, 10 µl of each *S. mutans* competing species was inoculated alongside the *S. gordonii* drop in a similar manner and allowed to incubate an additional 24 hr at 37°C in 5% CO_2_ before being photographed.

### 2.8 Hydrogen peroxide (H_2_O_2_) tolerance assay

To quantify the ability of the *cidB* mutant variants to tolerate exogenous H_2_O_2_, cells were challenged with 1 mM H_2_O_2_ in chemically defined FMC media. Cells were cultivated in FMC for 16–18 hr, then diluted to an OD_600_ of 0.02 in 3 ml fresh FMC. H_2_O_2_ (Fisher Scientific) was then added to a final concentration of 1 mM, and a 48‐well plate (Corning, Costar #3548) was inoculated with 500 µl of each strain in triplicate. OD_600_ was then measured at 2‐hr intervals over 24 hr using a Cytation 3 plate reader (BioTek, Vermont, USA) with incubation at 37°C.

### 2.9 Confocal microscopy and COMSTAT of static biofilms

In order to identify possible differences in biofilm formation between the *cidB* mutant variants, 5% CO_2_ and anaerobic biofilms were cultivated in BM media (Loo et al., 2000) containing 20 mM sucrose. Cells were first cultivated in BHI for 16–18 hr and then diluted to an OD_600_ of 0.02 in 3 ml fresh BM media. An optically clear 96‐well plate (Corning, Costar #3614) was then inoculated with 200 µl of each strain in triplicate. Biofilms were grown for 48 hr at 37°C either in atmospheric conditions supplemented with 5% CO_2_ or in a Pouch‐Anaero Anaerobic Gas Generating System for anaerobic growth. Biofilm culture supernatant was removed from each well prior to staining with the LIVE/DEAD BacLight Bacterial Viability Kit (Invitrogen, USA) using Syto‐9 (0.5 µl/ml) and propidium iodide (PI; 1.5 µl/ml) to detect live and dead/damaged cells, respectively. Imaging was performed on a Zeiss LSM 800 Confocal Light Microscope using ZEN software (Zeiss, Germany). Z‐stacks were generated using 0.5 µm slices at 63 × objective magnification with two random, center fields of view per well. Quantification of biofilm statistics was performed using COMSTAT (Heydorn et al., 2000) running on MATLAB R2010a (MathWorks) with manual thresholding on individual images collected on separate days.

### 2.10 Cell viability measurement of static anaerobic biofilms

To assay biofilm cell viability, UA159, *cidB* rough, and *cidB* smooth biofilms were cultivated in semidefined sucrose biofilm media in anaerobic pouches as described above. After 48 hr growth, growth media was removed, and biofilms were scraped and resuspended in sterile 0.85% NaCl. Serial dilutions were plated on BHI agar, and total CFUs were then enumerated after 48 hr growth at 37°C in a 5% CO_2_ incubator.

### 2.11 CSP competence assays

To assay the ability of the *cid* mutants to take up externally added plasmid DNA, a quantitative competence assay was performed using a previously published protocol (Seaton, Ahn, Sagstetter, & Burne, 2011) with the following modifications: *S. mutans* UA159 and isogenic mutant strains were grown in BHI broth for 16–18 hr at 37°C in a 5% CO_2_ incubator. Overnight cultures of each strain were diluted to an OD_600_ of 0.02 in BHI and then grown in a 96‐well plate to an OD_600_ of 0.13–0.15 before the addition of 81 ng plasmid DNA (methylated or unmethylated pOri23, as indicated in the results section), with or without addition of synthetic CSP (sCSP, Sigma‐Aldrich) to a final concentration of 0.5 µg/ml per culture. After 2.5 hr of further incubation at 37°C and 5% CO_2_, cultures were serially diluted and plated on BHI agar with and without selective antibiotic (erythromycin, 10 µg/ml). The number of colony‐forming units per milliliter (CFU/ml) of each culture was enumerated after 48 hr growth at 37°C in a 5% CO_2_ incubator. The transformation efficiencies were calculated as the percentage of transformants (CFU/ml on BHI + selective antibiotic) among total viable cells (CFU/ml on BHI).

### 2.12 XIP competence assays

Transformation efficiencies in chemically defined media were determined as described above, except that cultures were grown in defined FMC media (Terleckyj et al., 1975) and were supplemented with 1 µg synthetic SigX‐inducing peptide (sXIP, Sigma‐Aldrich) and 500 ng unmethylated pORI23 when cultures reached an OD_600_ of 0.135–0.15.

### 2.13 Statistical analysis

All statistical analyses were performed using SigmaPlot 13.0 (Systat Software). Data were tested for normality and equal variance prior to selection of appropriate parametric or nonparametric tests as indicated in each figure legend. Number of biological replicates analyzed in each experiment is specified in each figure legend.

## 3 RESULTS

### 3.1 The *cidB* mutant yields two stable colony phenotypes

**Figure 1:**
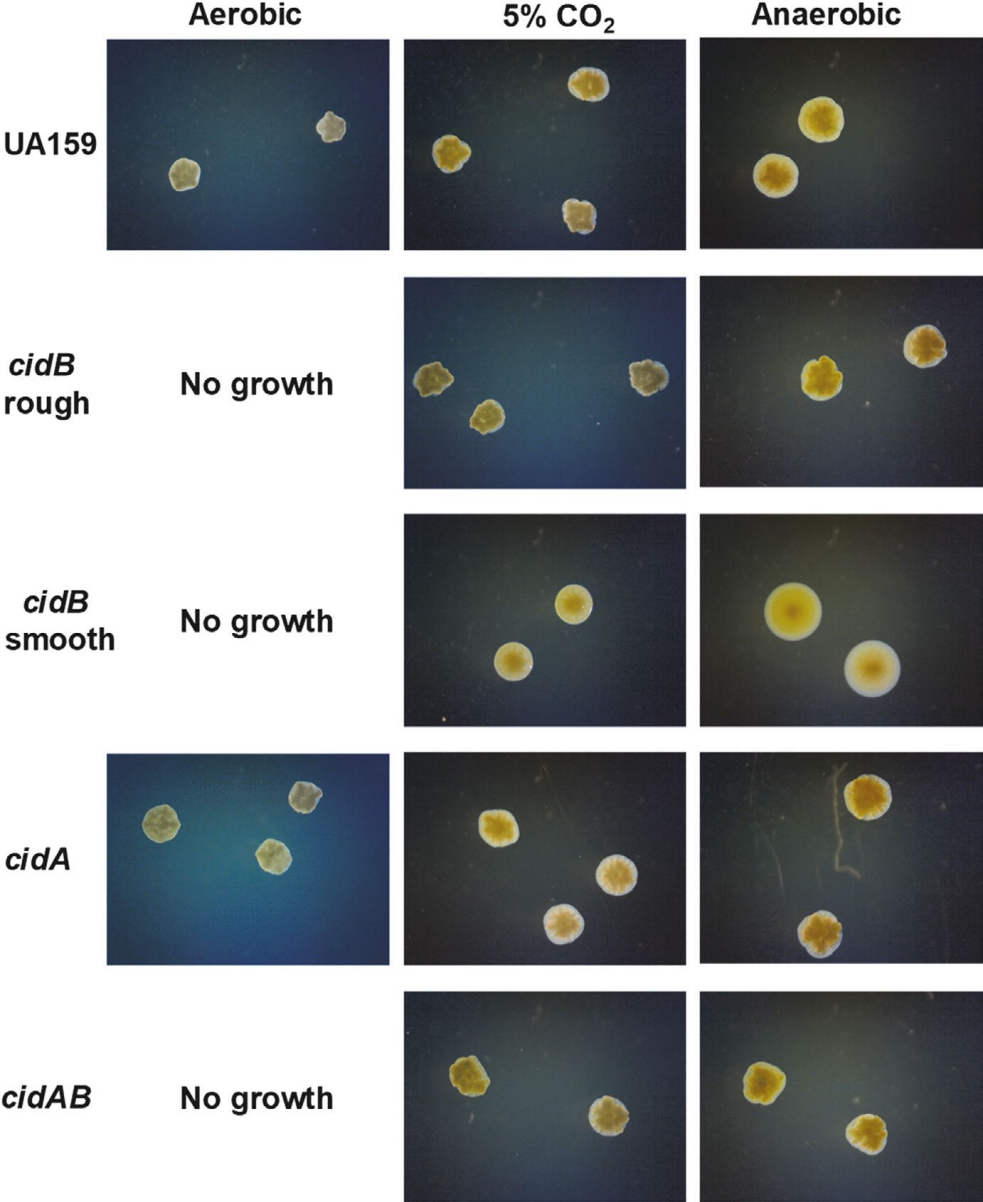
Influence of growth environment on *S. mutans* colony morphology. *S. mutans* wild‐type (UA159) and isogenic *cid* mutants were grown on BHI plates at 37°C for 48 hr in either normal atmospheric conditions (“aerobic”), 5% CO_2_, or in an anaerobic pouch. All images were taken at 10× magnification and are representative of *n* = 3 independent experiments

During routine culturing of *cidB* mutant frozen glycerol stocks, it was recently noted that two variant colony morphologies formed when struck out onto a BHI plate and grown in 5% CO_2_ supplemented conditions. One *cidB* variant was rough and granular matching the colony phenotype of the parental wild‐type strain *S. mutans* UA159, as well with isogenic *cidA* and *cidAB* mutants (Figure 1). The other *cidB* variant was observed to be smooth and round, lacking the granularity of both wild‐type and the *cidB* “rough” mutant (Figure 1). When grown anaerobically, this morphology repeated itself with the *cidB* “smooth” variant remaining round and mucoid while each of the other strains maintained their rough shape and granularity. When cultured in conditions with atmospheric levels of oxygen, both *cidB* colony variants, as well as the *cidAB* mutant, failed to grow (Figure 1), as was observed in our previous publication (Ahn et al., 2010). However, aerobic growth of the wild‐type and the *cidA* mutant displayed consistent colony morphology to that observed in anaerobic and CO_2_ supplemented growth conditions. Thus, the two *cidB* mutant variants were renamed: *cidB* rough for the granular variant and *cidB* smooth for the round, mucoid variant. Both *cidB* variant colony morphologies were deemed stable, as repeated subculturing from frozen glycerol stocks and colonies of *cidB* rough and *cidB* smooth always yield all rough or smooth colonies, respectively.

### 3.2 The *cidB* smooth mutant genome has lost TnSMU2 and neighboring genes

**Figure 2:**
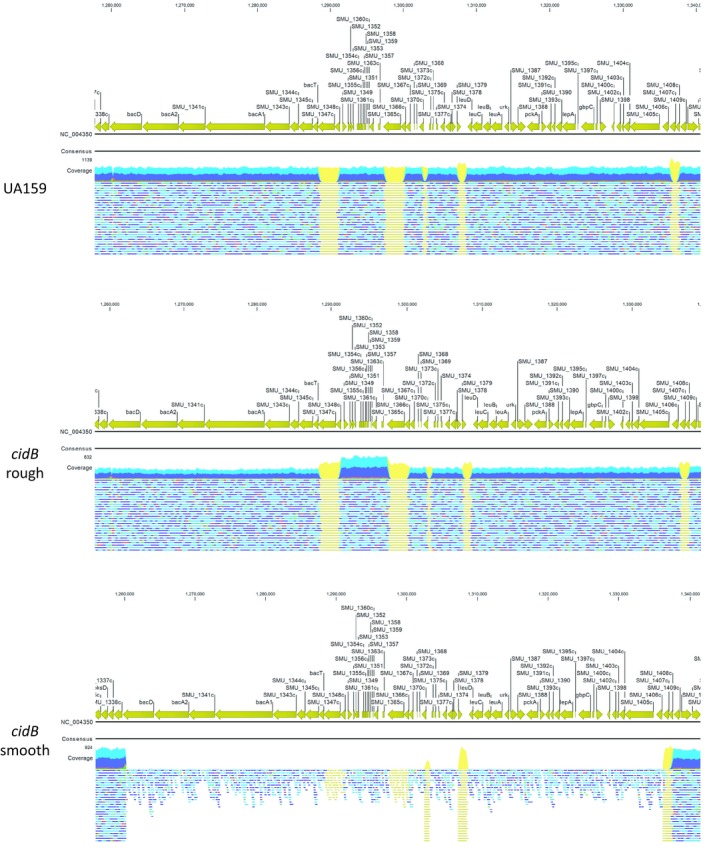
Wild‐type, *cidB* rough, and *cidB* smooth gDNA resequencing results displaying TnSMU2 and surrounding region. Mapped reads to TnSMU2 and surrounding area are displayed in green (forward only reads), red (reverse only reads), blue (read in both directions), and yellow (repetitive sequence motifs) to depth of 1,000 reads. Sequenced reads for *S. mutans* wild‐type (UA159, top) and the isogenic *cidB* rough variant (middle) show full presence of the TnSMU2 genomic island along with immediately neighboring regions. The corresponding genomic region of *cidB* smooth (bottom) displays significantly lower coverage for this region, as well as an approximately 20 kb section on the island's 3′ end

**Figure A1:**
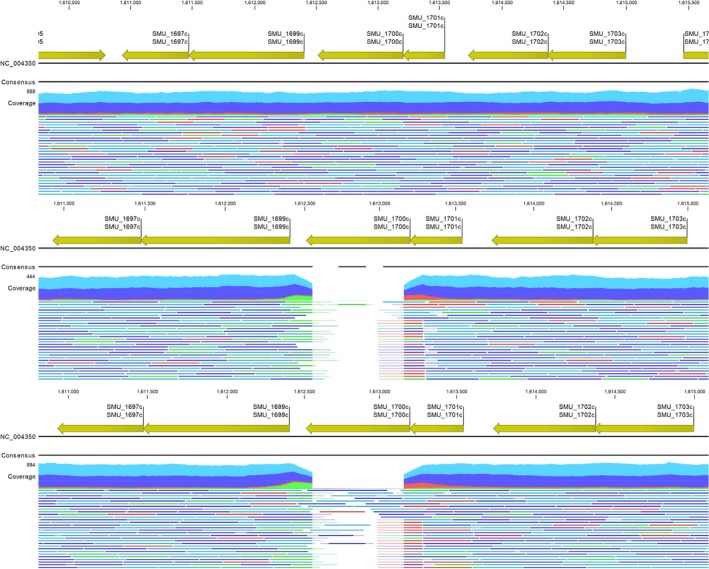
Wild‐type, *cidB* rough, *cidB* smooth gDNA re‐sequencing results displaying *cidAB* region. Mapped reads to *cidAB* surrounding area are displayed in green (forward only reads), red (reverse only reads), blue (read in both directions), and yellow (repetitive sequence motifs) to depth of 1,000 reads. Images are for *S. mutans* wild‐type (UA159, top), *cidB* rough (middle), and *cidB* smooth (bottom)

In order to determine what genetic variations may have led to altered colony morphologies of the *cidB* rough and *cidB* smooth variants, high depth whole genome resequencing was performed. Total gDNA was extracted from each of wild‐type UA159, *cidB* rough, and *cidB* smooth, which were then sequenced using a MiSeq Illumina Platform to a depth of 1,000 reads. Deletion of the *cidB* gene was confirmed in both *cidB* variants (Figure A1 in Appendix), with a small degree of aligned read noise attributed to *cidB*’s sequence homology to the *lrgB* gene (*SMU.575c*). Single nucleotide polymorphisms (SNPs) were identified based on differences from the *S. mutans* UA159 reference genome (NC_004350.2) and showed two SNPs within the wild‐type genomic resequencing that were not found in either *cidB* mutant (Table 2). SNP analysis of both *cidB* variants displayed many of the same SNPs as the isogenic wild‐type strain (Tables 2, A1, A2 in Appendix), but also presented four variations found in both *cidB* variants that were not present in the reference genome or our wild‐type strain (Table 1). Additionally, a major read coverage gap was identified in the *cidB* smooth mutant genome, corresponding to the TnSMU2 genomic island, in addition to ~20 kb of sequence on the region's 3′ end (Figure 2, bottom). This low‐coverage area starts at the beginning of TnSMU2, the gene *bacD* or *mubD* (Wu et al., 2010), and ends ~76kb downstream with *SMU.1406c* (a hypothetical protein of unknown function) (Figure 2). This entire area was found with full coverage in both the wild‐type and *cidB* rough strains (Figure 2, top and middle).

**Table 2:** Summary of SNP analysis

Strain	Nucleotide Change relative to published genome (NC_004350.2)	Overlapping annotation	Amino acid change
UA159 (SNP not present in either *cidB* mutant)	G → T	*fic, SMU.1207*	A78D
	G → C	*aspB*, *SMU.1312c*	A77G
*cidB* rough and *cidB* smooth (not present in UA159)	A → G	*SMU.12*	Y33C
	C → T	*pgi*, *SMU.307*	R258C
	A → G	*pgi*, *SMU.307*	E293G
	C → G	*dltA*, *SMU.1691c*	A466P

**Figure 3:**
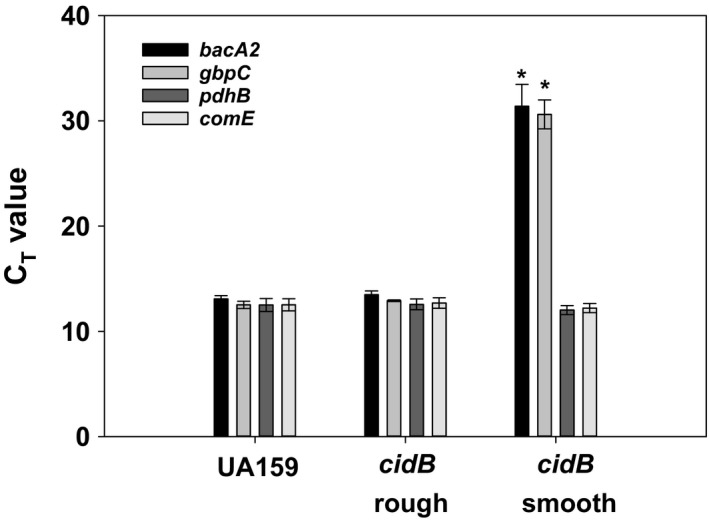
qPCR gene copy measurements of TnSMU2‐related genes. Relative copy numbers were determined by qPCR using wild‐type (UA159), *cidB* rough*,* and *cidB* smooth genomic DNA. The C_T_ value represents the qPCR cycle at which detectable amplification occurred for each gene product. Data represent the average of *n* = 3 (wild type, *cidB* rough) or *n* = 4 (*cidB* smooth) independent experiments; error bars display standard deviation with * denoting statistical significance relative to wild type (Dunn's test (*bacA2*) or Holm–Sidak (*gbpC*), *p* < .05)

Given that coverage of the TnSMU2 region by sequence reads was not completely absent in the *cidB* smooth mutant, qPCR was also performed on gDNA isolated from wild type, *cidB* rough, and *cidB* smooth, using primers specific for genes both within and outside the “low‐read coverage” TnSMU2 region of the *cidB* smooth strain. Primers were generated for two genes inside of the low‐coverage region, *bacA2* and *gbpC*, as well as two genes outside of the region, *pdhB* and *comE* (Table 1). Relative copy numbers were determined as a function of the C_T_ values generated by each qPCR, with higher C_T_ values indicating lower levels of initial gDNA template copy number available for amplification. Analysis of C_T_ values for those genes outside of the TnSMU2 region indicated similar abundance for each of *pdhB* and *comE*, with little difference in C_T_ values noted between each of the three strains (Figure 3). C_T_ values for *bacA2* and *gbpC* products were also comparable between wild type and *cidB* rough, with mean C_T_ values of ranging from 12 to 13. In the *cidB* smooth variant however, these C_T_ values were significantly higher, ranging from 30 to 31 for *gbpC* and *bacA2* products (Figure 3). Although these qPCR data correlate with the sequencing read coverage patterns observed in wild type, *cidB* rough, and *cidB* smooth (Figure 2), it is not clear whether the TnSMU2 genomic region is in very low abundance or completely absent in the *cidB* smooth variant.

### 3.3 Both *cidB* mutant variants are more sensitive to oxidative stress but display comparable acid tolerance relative to wild type

**Figure 4:**
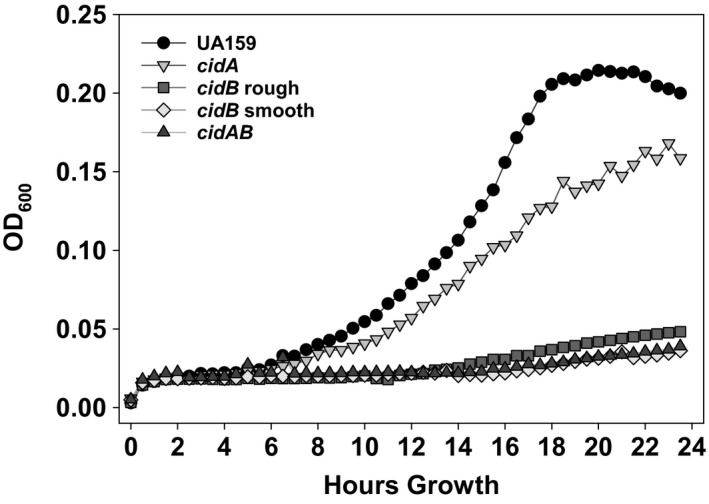
Influence of *cid* mutations on aerobic growth. Growth curves of wild‐type *S. mutans* (UA159) and the *cid* panel of mutants under aerobic challenge. Cells were grown in FMC media using an automated Bioscreen C growth curve system with constant shaking at 37°C. Data are the representatives of *n* = 3 independent experiments

As a defect in oxidative stress tolerance had been previously observed in the *cidB* rough mutant (Ahn & Rice, 2016), we proceeded to compare each *cidB* variant's ability to survive aerobic growth conditions relative to wild type and other *cid* mutants. When grown in defined FMC media with constant shaking, the *cidA* mutant displayed an increased lag phase compared to wild type with a lower final cell density after 24 hr (Figure 4). Growth of the *cidAB* mutant and both *cidB* mutant variants was severely impaired in this assay, as all three strains failed to rise above an OD_600_ of 0.05, remaining at baseline at timepoints when the *cidA* mutant entered exponential growth (10 hr) or reached peak optical density (24 hr) (Figure 4). A small, linear increase in OD_600_ was noted in *cidB* rough starting at 15 hr growth, but this increase was minimal.

**Figure 5:**
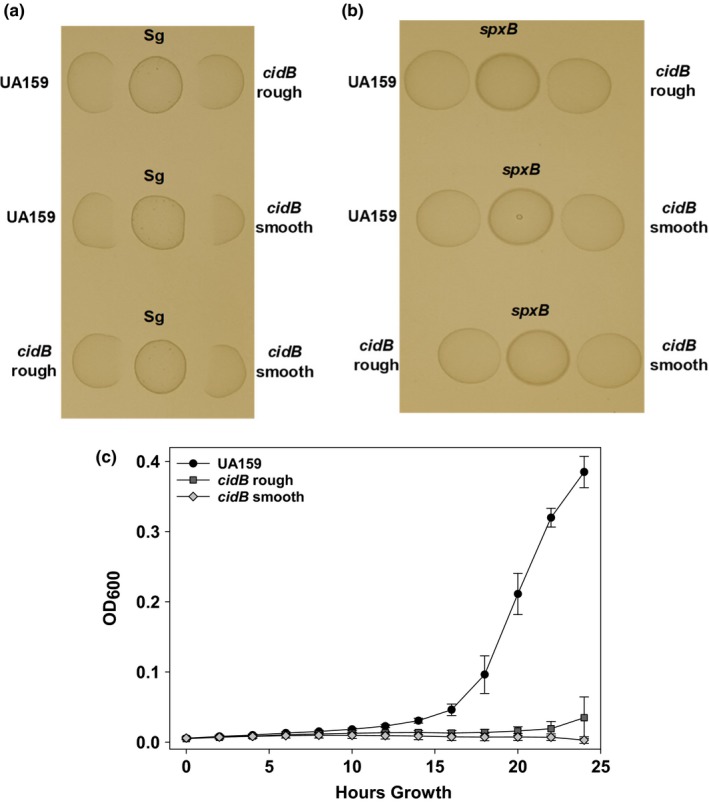
Dot drop competition between *S. mutans cidB* mutants and *S. gordonii,* and quantitative H_2_O_2_ challenge assay. The ability of *S. gordonii* wild‐type (Sg) (a) and isogenic *spxB* mutant (b) to inhibit *S. mutans* wild‐type (UA159) and isogenic *cidB* mutant growth was assessed using a dot drop competition assay as described in Materials and Methods. Data represent *n* = 3 independent experiments. The ability to grow planktonically in FMC containing 1 mM H_2_O_2_ was also quantified in all three strains by OD_600_ measurements over a 24‐hr period (C). Data represent *n* = 3 independent experiments, error bars = *SEM*

**Figure A2:**
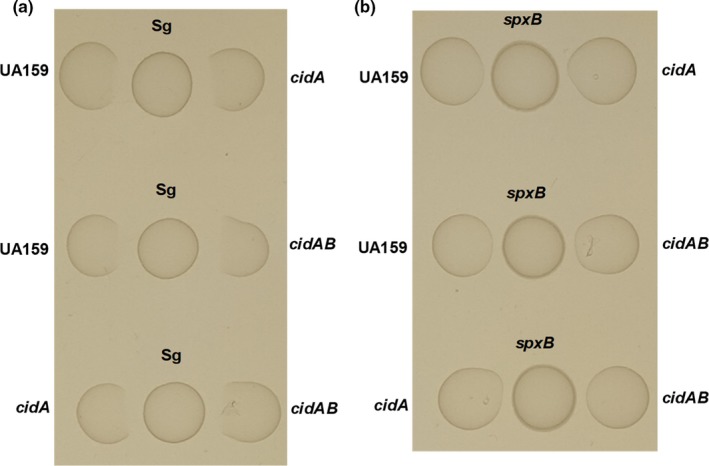
Dot drop competition between *S. mutans cid* mutants and *S. gordonii*. The ability of *S. gordonii* wild‐type (Sg) (a) and isogenic *spxB* mutants (b) to inhibit *S. mutans* wild‐type (UA159) and isogenic *cid* mutant growth was assessed using a dot drop competition assay as described in Materials and Methods. Data represent *n* = 3 independent experiments

In order to explore possible oxidative stress tolerance differences between the *cidB* variants with a peroxygenic competitor, dot drop competition assays were then performed with the oral commensal *S. gordonii*. Growth inhibition of all *S. mutans* strains in this assay was observed when challenged with wild‐type *S. gordonii* (Figure 5a, top and middle). However, the *cidB* smooth mutant appeared to display greater growth inhibition compared with wild type and *cidB* rough (Figure 5a, middle and bottom). In contrast, inhibition of *cidA* and *cidAB* mutants by wild‐type *S. gordonii* was more modest and similar to *S. mutans* UA159 (Figure 2a in Appendix). Growth inhibition was rescued for all strains when challenged with a pyruvate oxidase (*spxB*) mutant strain (defective in H_2_O_2_ production) of *S. gordonii* (Figure 5b and Figure A2b in Appendix). In a confirmatory experiment, wild‐type variant and each *cidB* mutant variant were also grown planktonically in the presence of 1 mM H_2_O_2_ (Figure 5c), which demonstrated that both *cidB* mutants displayed comparable growth inhibition relative to the wild‐type strain. Collectively these results suggest that although both *cidB* variants are comparably deficient in tolerating planktonic aerobic growth and chemical 1 mM H_2_O_2_ challenge (Figures 4, 5c), the *cidB* rough mutant may be better able to tolerate H_2_O_2_ stress generated by *S. gordonii* on BHI agar plates relative to *cidB* smooth, presumably due to its intact TnSMU2 locus.

**Figure A3:**
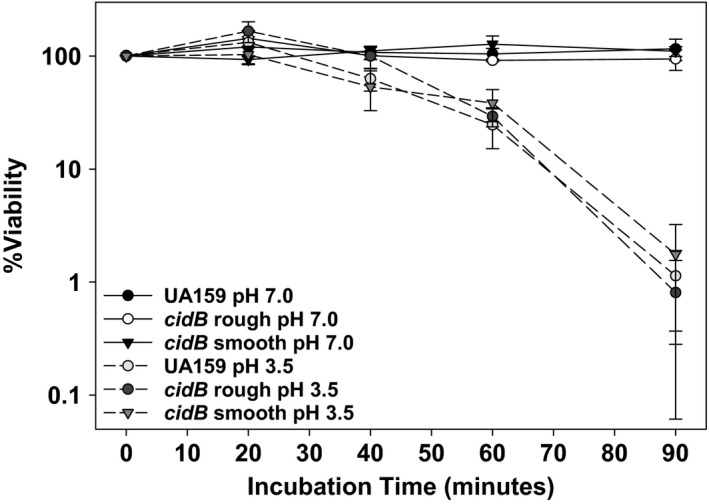
Influence of *cidB* mutation on acid tolerance. Viability of late‐exponential phase wild‐type (UA159), *cidB* rough, and *cidB* smooth mutants in response to acid stress (pH 3.5 buffer) and neutral pH buffer (pH 7.0) over a 90‐min period was assessed by serial dilution plating and CFU counts. Viability was expressed as a percentage of the CFU/ml at each time point divided by the CFU/ml of *t* = 0 (when cells first transferred to treatment buffer) for each strain and treatment. Data represent the average of *n* = 3 independent experiments, error bars = *SEM*

The ability for each *cidB* mutant variant to tolerate acid stress was also assessed by challenging wild type and each strain to pH 3.5 (Figure A3 in Appendix). In this experiment, all strains displayed comparable decreases in cell viability over time in the pH 3.5 treatment condition (~99% loss of viability by 90 min) and demonstrated similar levels of survival in the pH 7.0 control condition. These results suggest that neither CidB nor TnSMU2 is required for acid tolerance under the conditions tested in this study.

### 3.4 Both *cidB* and the *TnSMU2* genomic region influence *S. mutans* biofilm structure

**Figure 6:**
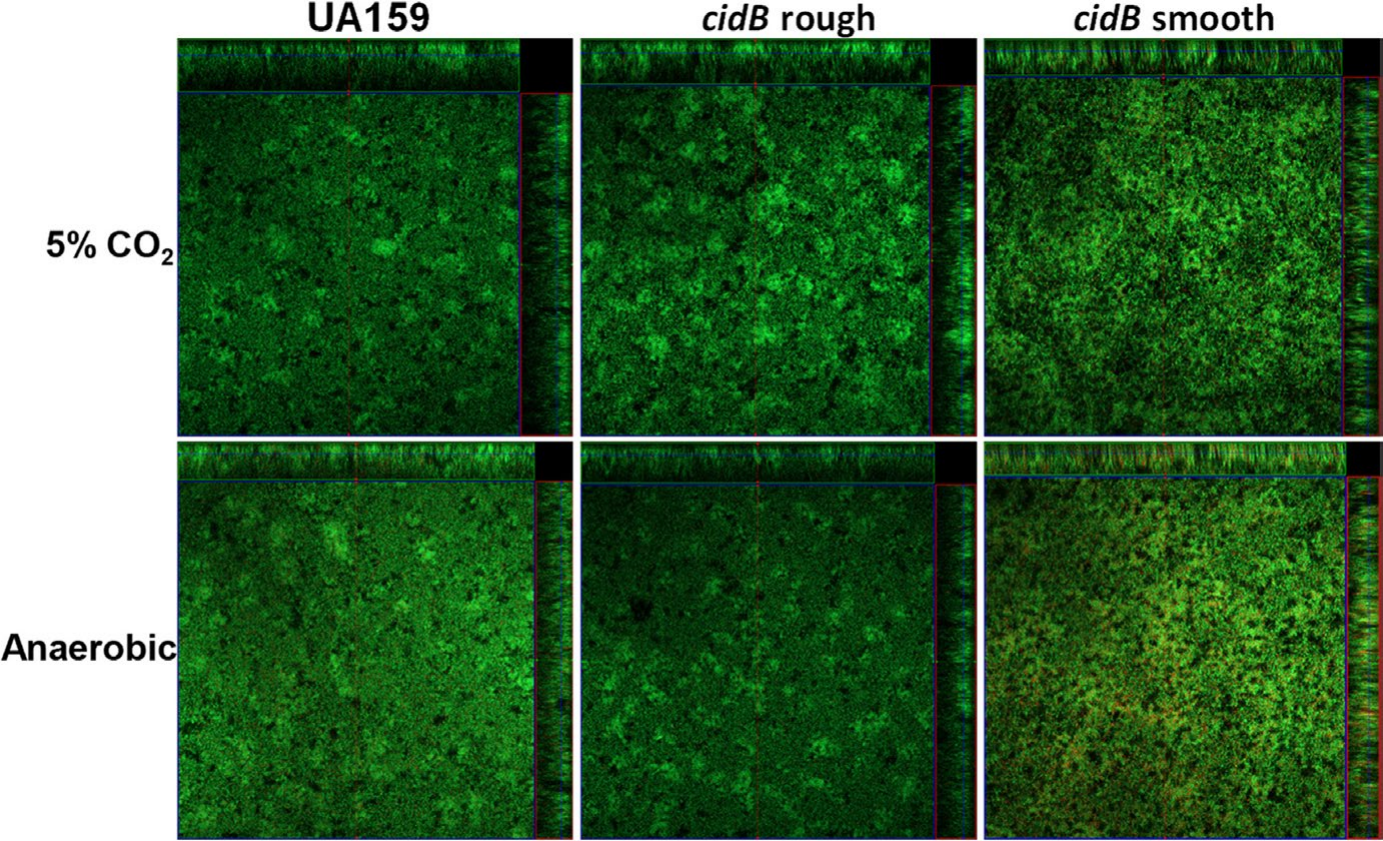
Representative biofilm images of wild‐type, *cidB* rough, and *cidB* smooth mutants. Confocal microscopy images of wild‐type, *cidB* rough, and *cidB* smooth biofilms grown for 48 hr in semidefined media with 20 mM sucrose. Biofilms were stained for viable (green) or dead/damaged (red) using Syto‐9 and propidium iodide, respectively. Images show the orthogonal view of the biofilm centered on a Z‐stack in the first third and are representative of *n* = 18–27 random fields of view taken over *n* = 3–5 independent experiments

Biofilm formation is another key physiological process of *S. mutans* which has been previously described as impacted by the Cid/Lrg system (Ahn et al., 2010). Both CO_2_ and anaerobic growth conditions were chosen in order to study the alterations in biofilm formation due to the presence of oxygen. Visualization of each biofilm by LIVE/DEAD staining (Figure 6) revealed a full, healthy looking wild‐type biofilm consisting primarily of live (green) cells (Figure 6, left). The anaerobic wild‐type biofilm appeared thicker in the horizontal cross‐sectional view, with many of the holes or pockets in the CO_2_ biofilm (Figure 6, top left) replaced with a more homogenous mat of cells (Figure 6, bottom left). The *cidB* rough mutant appeared to have decreased biofilm production under anaerobic growth conditions (Figure 6, middle), with a noted reduction of cell mass seen in the biofilm cross sections. Increased red/yellow signal (as a result of PI staining) was observed in the *cidB* smooth anaerobic biofilm (Figure 6, bottom right) indicating an increased presence of dead and/or damaged cells within the biofilm matrix. The *cidB* smooth CO_2_ biofilm also appeared to have decreased thickness, as also observed in both the top‐down and cross‐sectional views (Figure 6, right). An interesting honeycomb‐like pattern was also observed in both growth conditions for the *cidB* smooth mutant (Figure 6, right).

**Figure 7:**
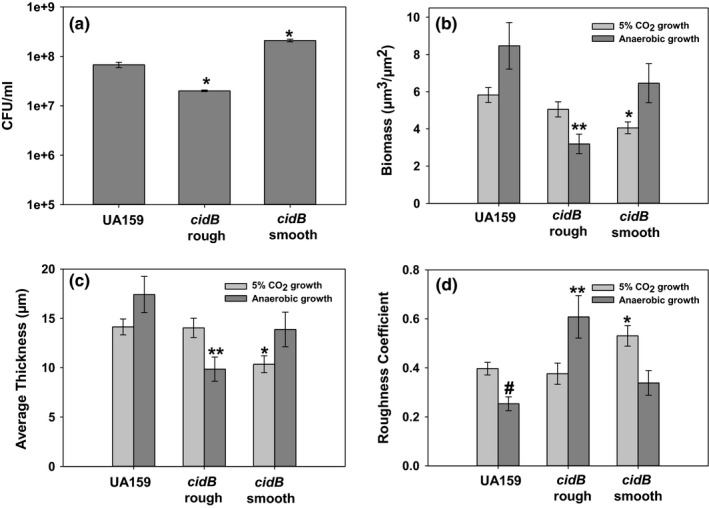
CFU and COMSTAT analysis of wild‐type, *cidB* rough, and *cidB* smooth biofilms. Quantification of viable biofilm cells by CFU serial dilution plating (a) was performed on 48 hr UA159 (wild‐type), *cidB* rough, and *cidB* smooth anaerobic biofilms. Data represent *n* = 3 biological replicates, error bars = *SEM*. * represents significant difference compared to wild type (*p* < .01, Holm–Sidak). Total biomass (b), average biofilm thickness (c), and the roughness coefficient (d) were quantified using pixel density and calculated through the COMSTAT algorithm (Heydorn et al., 2000) in MATLAB. The roughness coefficient represents the heterogeneity of the biofilm surface, with higher values indicative of a less uniform surface. Light bars represent biofilms generated during CO_2_ growth, and dark bars represent biofilms generated during anaerobic conditions. Data represent the average of *n* = 18–27 random fields of view acquired over *n* = 3–5 independent experiments. Error bars = standard error of the mean (*SEM*), * represents significant difference between CO_2_ growth conditions compared to wild type (*p* < .05, Holm–Sidak for biomass and thickness, Dunn's test for Roughness coefficient), ** represents significant difference between anaerobic growth conditions compared to wild type (*p* < .05, Dunn's test), # represents significant difference between anaerobic wild type and CO_2_ wild type (*p* < .001, Mann–Whitney rank sum test)

To quantify viability of the anaerobic 48 hr biofilms, CFU plating was performed in a parallel experiment (Figure 7a). Although increased PI staining was observed in the *cidB* smooth mutant (Figure 6, bottom right), this did not translate to decreased viability as assessed by CFU counts, and in fact, CFU counts were significantly higher in the *cidB* smooth mutant compared to wild type (Figure 7a), which could indicate that its increased population of PI‐stained cells represents a damaged but viable subpopulation of the biofilm. Interestingly, a significantly decreased level of viable cells was observed by CFU counts in the *cidB* rough mutant compared to wild‐type (Figure 7a). COMSTAT analysis of wild‐type and both *cidB* mutant biofilms was also performed to quantify biofilm parameters of each strain (Figure 7b‐d). These analyses revealed a modest trend in increased biofilm biomass and thickness for wild type during anaerobic growth compared with CO_2_ growth, but these results were not statistically significant (*p* > .05, Mann–Whitney rank sum test, Figure 7b, c). The opposite trend was noted however with the roughness coefficient (Figure 7d), whereby wild‐type anaerobic biofilm values were significantly reduced relative to wild‐type CO_2_ biofilms (*p* < .001, Mann–Whitney rank sum test), suggesting that anaerobic wild‐type biofilms are more homogenous than CO_2_ grown samples. The c*idB* rough mutant biofilms were closely matched to wild type in terms of biomass, thickness, and roughness coefficient in the CO_2_ growth condition, but anaerobic *cidB* rough mutant biomass and thickness were reduced almost twofold compared with wild‐type under this same condition (Figure 7b, c), which correlates with the decreased CFU counts observed in this strain (Figure 7a). An almost threefold increase in roughness coefficient was also observed in the anaerobic *cidB* rough biofilm relative to wild‐type (Figure 7d). In contrast to *cidB* rough, the *cidB* smooth mutant biofilms displayed significantly (*p* < .05, Holm–Sidak Test) decreased average biomass and thickness during CO_2_ growth compared with wild‐type (Figure 7b, c), and a significantly increased (*p* < .05, Dunn's test) roughness coefficient (Figure 7d). Biofilm growth of the *cidB* smooth mutant under anaerobic conditions minimized these differences compared with wild‐type. Qualitative examination of these images (Figure 6), along with COMSTAT quantification of biofilm metrics, suggests that both *cidB* mutant variants have altered biofilm properties that are observed in opposite growth conditions. Given that the increased cell PI staining phenotype was unique to the *cidB* smooth mutant, it is likely that this is associated with genomic loss of TnSMU2 and/or neighboring genes.

### 3.5 Mutation of *cidB* and loss of TnSMU2 correlate with altered competence

**Figure 8:**
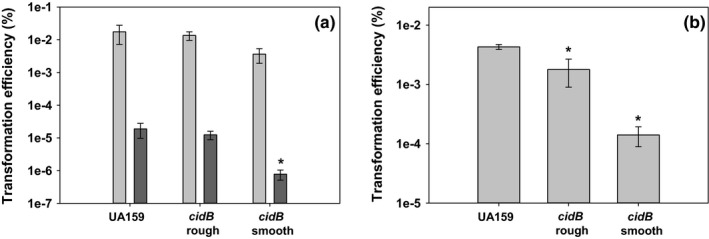
CSP (a) and XIP (b) competence assays. (a) Transformation efficiencies of wild type (UA159), *cidB* smooth, and *cidB* rough in complex BHI media. Light bars depict efficiencies with added sCSP, and dark bars depict efficiencies without sCSP. Data represent *n* = 5 independent experiments. Error bars represent *SEM*, with * denoting statistical significance (*p* < .05, Student–Newman–Keuls test) compared to wild type. (b) Transformation efficiencies of *S. mutans* strains in defined FMC media supplemented with sXIP. Data represent *n* = 3–4 independent experiments. Error bars represent *SEM*, with * denoting statistical significance (*p* < .05, Holm–Sidak test) compared to wild type

**Figure A4:**
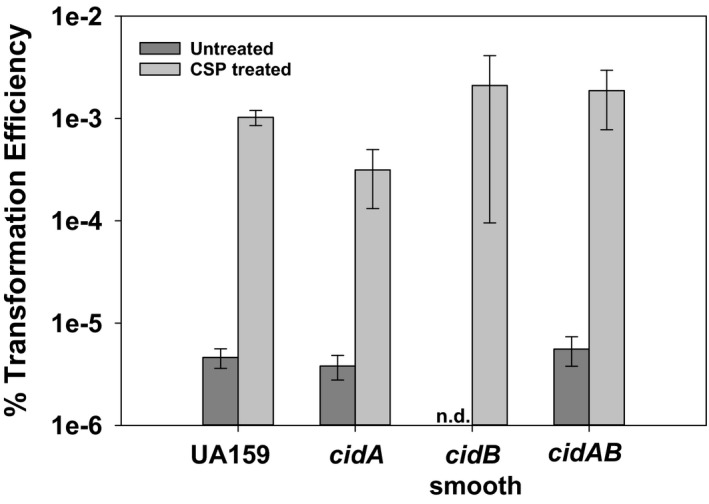
CSP competence assays. Transformation assay and efficiencies of wild‐type (UA159) and isogenic *cid* mutants were performed and calculated as described in Materials and Methods, except that methylated pOri23 plasmid was used. Data represent *n* = 3–4 independent experiments. Error bars represent *SEM*, and n.d. indicates that transformation efficiencies were not determined, as no colonies were observed on antibiotic plates

We had previously observed that mutation of *lrgA* leads to CSP‐related competence deficiency (Ahn, Qu, Roberts, Burne, & Rice, 2012). Therefore, the influence of *cid* genes on the ability of *S. mutans* to uptake foreign DNA was tested in this study using quantitative competence assays in both complex and defined media. Both media were tested in order to probe both the CSP‐ and XIP‐mediated competence systems found within *S. mutans*. With addition of synthetic CSP (sCSP), each of the wild type and *cid* mutants had similar transformation efficiencies (Figure 8 and Figure A4 in Appendix) with *cidB* smooth being slightly lower than wild type and the *cidB* rough variant (Figure 8a). As expected, transformation efficiencies without the addition of sCSP were dramatically lower for all strains. However, the *cidB* smooth transformation efficiency was significantly lower (~1 log reduction, *p* < .05, Student–Newman–Keuls test) compared with the wild‐type strain and *cidB* rough (Figure 8a). Mutation of either *cidA* or *cidAB* did not alter transformation efficiency in BHI media in the presence or absence of sCSP (Figure A4 in Appendix).

When grown in defined media and supplemented with synthetic XIP (sXIP), distinct phenotypes were observed with each *cidB* variant. The transformation efficiency of the *cidB* rough variant was modestly decreased compared with wild type (Figure 8b; ~0.5 log decrease, *p* = .014, Holm–Sidak Test), but the *cidB* smooth mutant transformation efficiency was significantly lower with a ~1.5 log decrease in transformation efficiency compared with wild‐type (*p* < .001, Holm–Sidak Test) and a ~1 log difference compared with *cidB* rough (*p* = .043, Holm–Sidak Test). These results indicate that deletion of *cidB* also negatively impacts *S. mutans* genetic competence during growth in defined media, but the loss of TnSMU2 and/or neighboring genes increases this effect significantly.

## 4 DISCUSSION

In order to successfully persist and cause disease within the oral cavity, *S. mutans* must be able to survive numerous environmental stresses. The *cidB* gene has been shown to be an important contributor to the oxidative stress response of *S. mutans* with wide‐ranging effects on expression of numerous metabolic genes and the GI TnSMU2 (Ahn & Rice, 2016). Our characterization of each *cidB* mutant variant has both confirmed the role of *cidB* in *S. mutans* physiology and reinforces a possible link between this gene and TnSMU2. However, at this time the exact mechanism by which the *cidB* smooth variant emerged is not known and is currently under investigation. We had previously attempted an initial evolution experiment to determine the frequency of stable smooth colony formation from repeated passaging of both wild‐type and *cidB* rough strains on BHI plates under aerobic, CO_2_, and anaerobic growth conditions. After 50 passages for *cidB* rough and 14 passages for wild type, we were unable to detect the appearance of any stable smooth variant colonies from either strain. Given that the original stock culture of *cidB* rough appears to have a relatively equal mixture of smooth and rough colonies (the subculturing of each yielding a stable phenotype), it is possible that the *cidB* smooth variant arose during the initial transformation to generate the *cidB* mutant. On‐going and future research efforts include a careful assessment of the frequency of stable smooth colony phenotype after transformation of wild type, whether this frequency is increased when *cidB* is absent and genomic loss of TnSMU2 always correlates with emergence of stable smooth colony variants.

In the *cidB* smooth variant, the only genomic sequence difference of note, compared with *cidB* rough, is low‐read coverage within and 3′ to TnSMU2. This region contains upwards of 60 coding sequences, though many are annotated as transposon fragments or repetitive sequences. Many are hypothetical with several other genes annotated to be involved in metabolism (especially isopropylmalate synthesis, *SMU.1384*) or the CRISPR1/Cas system with Cas1, Cas2, and Cas9 located within an operon in this region (*SMU.1402* through *SMU.1405*) next to a CRISPR array spanning several ORFs (Serbanescu et al., 2015). Genes within this region, as well as the CRISPR2/Cas loci (*SMU.1753c*‐*SMU.1755c*, *SMU.1757c* & *SMU.1758c*, and *SMU.1760c* through *SMU.1764c*), were also among those whose expression was affected by a deletion of *lrgAB* (Rice, Turner, Carney, Gu, & Ahn, 2017). Taken alongside the strikingly similar expression transcriptomic profiles of the *cidB* rough mutant (Ahn & Rice, 2016), these data suggest a close link in the cellular role(s) of Cid and Lrg with TnSMU2 and bacterial cell immunity through CRISPR/Cas. Additional evidence of connections between Cid/Lrg and TnSMU2 were also uncovered during microarray analysis of transcriptomic changes in a *lytS* mutant, which encodes the sensor kinase of the LytST two‐component system that serves as a primary regulator of *lrgAB* expression (Ahn et al., 2012). Genes within TnSMU2 were again among the most affected by a mutation of *lytS*, with expression significantly downregulated compared with wild type. Expression of TnSMU2 genes was conversely increased in both the *lrgAB* and *cidB* mutants (Ahn & Rice, 2016; Rice et al., 2017), which may indicate that function of these gene products is suppressive in some way to expression of the TnSMU2 region. Possibly, regulation of *lrgAB* by LytS balances this negative effect.

Change in colony morphology due to loss of the TnSMU2 region may not be from the loss of any one of these ORFs, but the collective loss of all related genes may cause larger physiological changes that alter the appearance of colonies on an agar plate. It is also important to note that TnSMU2 is not found in all *S. mutans* strains (Lapirattanakul et al., 2008; Wu et al., 2010) and that many *S. mutans* backgrounds have differing colony morphologies (Emilson, 1983). Previous work in other serotype *c S. mutans* isolates has identified large, mucoid colonies similar in description to the *cidB* smooth strain, and characterized this alteration as a result of altered fructosyltransferase (FTase) activity (Okahashi, Asakawa, Koga, Masuda, & Hamada, 1984). Although we have not identified any genes within this region predicted to take part in fructan synthesis, the possibility remains that one of the many hypothetical proteins may indeed have a role in this process.

Due to the presence of low‐read coverage within the TnSMU2 region of the *cidB* smooth mutant genome, we are not able to conclusively state this region has been completely lost. In order to better understand if genes within this region were present, as well as to quantify the gap in coverage, qPCR primers were targeted to genes within the region. These results confirmed that copy numbers of *comE* and *pdhB*, two genes outside of the low‐coverage area, are consistent throughout our wild‐type *S. mutans* and both *cidB* mutant variants. This was expected, as neither gene showed gaps in coverage during our sequence analysis even though *pdhB* resides on the edge of the 3′ end of the low‐coverage region. However, relative quantities for *bacA2* and *gbpC* differed in this qPCR analysis: Both genes were present in comparable relative copy number between wild‐type and *cidB* rough genomic DNA (as indicated by near‐identical C_T_ values to each other, which were also in line with C_T_ values observed for *pdhB* and *comE* for all three strains). However, *bacA2* and *gbpC* C_T_ values in the *cidB* smooth strain were very high (~30), indicating a low copy number relative to wild type and *cidB* rough. Although qPCR amplification of these genes was detectable in *cidB* smooth, their C_T_ values approach the qPCR cutoff which is normally considered background (C_T_ ≥ 35). This result alone still does not clarify whether or not this region is completely absent in the *cidB* smooth genome, or whether the TnSMU2 region is somehow being maintained in a small subpopulation of this strain. Experiments are currently in progress to distinguish between these two scenarios, as well as to probe the frequency of loss of TnSMU2 in *cidB* mutants in both previously and newly constructed mutants.

Two vital physiological processes for *S. mutans* persistence within the oral cavity are biofilm formation and the ability to withstand oxidative stress (Kreth et al., 2005; Tsumori & Kuramitsu, 1997). While acid tolerance was not shown to be affected in either *cidB* mutant variant (Figure A3 in Appendix), mutation of *cidB* has previously been shown to have a significant effect on oxidative stress tolerance in *S. mutans* grown in BHI (Ahn & Rice, 2016) and on agar plates in atmospheric levels of O_2_ (Ahn et al., 2010). Congruent with these results, neither *cidB* variant nor the *cidAB* mutant grew successfully in atmospheric aerobic conditions planktonically (Figure 4), or on agar plates (Figure 1). The *cidA* mutant however was able to grow comparatively well in defined media during aerobic growth. Competing each *cidB* variant against *S. gordonii* did reveal a modest separation of these two mutants in terms of H_2_O_2_ resistance when cultured on BHI agar plates (Figure 5a). The *cidB* smooth mutant appeared to display greater inhibition by wild‐type *S. gordonii,* with a larger area of obstructed growth as directly compared with either the *cidB* rough variant or wild‐type (Figure 5a). This phenotype was lost when competed against a *S. gordonii spx* mutant which is defective in H_2_O_2_ production, confirming this inhibition was a function of H_2_O_2_ generated by *S. gordonii* rather than production of an excreted secondary metabolite or peptide. Increased inhibition of *cidB* rough as compared with wild type was also observed, suggesting that *cidB* itself is important for competitive fitness within the oral cavity, but loss of the TnSMU2 region in *cidB* smooth may contribute to its increased growth inhibition by H_2_O_2_‐producing *S. gordonii* on BHI agar plates. Deletion of the *bac/mub* operon (encoding a nonribosomal peptide synthetase‐polyketide synthase gene cluster responsible for pigment biosynthesis) within TnSMU2 has been previously shown to increase sensitivity to oxidative stress in three different *S. mutans* strain backgrounds (Wu et al., 2010). Therefore, it is likely that loss of these genes in the *cidB* smooth mutant contributes to its increased growth inhibition by H_2_O_2_‐producing *S. gordonii*. Defining a specific stress‐related effect of elimination of this region is made even more complex due to the variability of this region seen in *S. mutans* clinical isolates characterized thus far (Lapirattanakul et al., 2008; Wu et al., 2010).

Assessment of biofilm formation between the *cidB* variants also revealed an interesting effect of this gene on the structure and amount of biofilm generated by *S. mutans*. During growth in CO_2_ supplemented conditions, biofilms produced by *cidB* rough closely resembled those of wild type, whereas biofilms produced by *cidB* smooth were significantly diminished with lower biomass and average thickness under this same growth condition. This *cidB* smooth mutant biofilm phenotype may be driven in part by the loss of the glucan binding protein *gbpC*, which is not a part of TnSMU2 but was part of the 3′ end missing from *cidB* smooth (Figure 2). Biofilm growth of *cidB* smooth under anaerobic conditions reversed the phenotypic effects observed in CO_2_ growth. In comparison, biofilms formed by the *cidB* rough mutant were altered during anaerobic growth (decreased biomass and thickness) but not during CO_2_ growth, suggesting that loss of *cidB* alone leads to a reduction of anaerobic biofilm formation, while concurrent loss of the TnSMU2 region may compensate this biofilm reduction in some manner. Viable cell counts within each anaerobic biofilm reflected these findings, as the *cidB* rough mutant displayed a significantly lower cell count than either the wild type or *cidB* smooth. Indeed, the smooth variant anaerobic biofilm displayed the highest viable cell count of all three tested strains despite the increase in PI staining observed in the confocal images (Figure 6). The function of *cidB* may thus be tied to *S. mutans* anaerobiosis and persistence. Loss of the large coding sequences within the TnSMU2 region may also reduce the overall carbon requirements for *S. mutans* in the *cidB* smooth mutant during anaerobic growth, possibly allowing it to maintain wild‐type levels biofilm formation despite increased presence of cell damage.

Reductions in genetic competence can prevent horizontal gene transfer within the oral microbiome, disabling transfer of antibiotic resistance genes (Hannan et al., 2010; Li, Lau, Lee, Ellen, & Cvitkovitch, 2001), disrupting acquisition of MGEs (Ciric, Mullany, & Roberts, 2011), and/or decreasing subpopulation heterogeneity and overall fitness of the *S. mutans* population. In this study, a significant decrease in the ability for both *cidB* variants to uptake plasmid DNA was shown in defined media where the XIP‐mediated ComRS system is predominant. The *cidB* smooth variant was less able to uptake added plasmid DNA compared with *cidB* rough and also displayed a deficiency in plasmid DNA uptake in complex media. This phenotype was rescued however with the addition of sCSP. As ComRS‐mediated competence activation directly stimulates *comX*, observed competence defects in both *cidB* mutant variants indicate a relationship between *cidB* and the ability of *S. mutans* to activate *comX*. This effect is not observed in complex media however, indicating that diminished competence in *cidB* smooth is also contingent on loss of the TnSMU2 region. Genes impacting natural competence have been mapped across the *S. mutans* genome via transposon sequencing (Shields et al., 2018) and includes *SMU.1398*, annotated *irvR* as a phage‐associated repressor protein. Found within the reduced TnSMU2 region, losing this gene may contribute to the competence defect observed in defined media as well as explain the *cidB* smooth competence defect in complex media. A study by Khan et al. demonstrated the expression of many genes located within the TnSMU2 region and its 3′ end to be significantly altered in cultures treated with CSP after 10 or 100 min (Khan et al., 2016). Some of these genes, such as the hypothetical 3‐isopropylmalate dehydrogenase *SMU.1383*, displayed increased expression in the wild‐type background at both timepoints, but decreased expression in a *comS* mutant. Others, such as *gbpC*, displayed consistent expression profiles in all three treatments with increased expression throughout. While several were stated to be statistically significant, many of these alterations failed to clear a mean fold change cutoff of 2.0. Regulation of competence is incredibly complex; thus, alterations of expression for many of these genes may be downstream effects from more significant changes in other loci. Further experiments beyond the scope of this current study are necessary to determine what the causal agent of the observed competence defects is in both *cidB* variants or if these competence defects derive directly from the loss of TnSMU2 and its downstream region.

While the precise function of CidAB remains a mystery, this study has reinforced a link between these genes and the genomic island TnSMU2. Our data also provide novel insight into the physiological role CidB plays within the cell, in addition to how the presence or absence TnSMU2 affects *S. mutans* physiology. Determining the importance of *cidB* and TnSMU2 in *S. mutans* oxidative stress tolerance, biofilm production, and competence helps provide new insights as to how this bacterium is able to persist within the oral cavity and cause disease, while also providing new evidence in regard to the function of these relatively uncharacterized areas of *S. mutans* physiology.

## CONFLICT OF INTEREST

None declared.

## AUTHOR CONTRIBUTIONS

MET, SJA, and KCR were involved in conceptualization; MET, KH, OVC, DG, and RKC were involved in investigation; MET, KH, RKC, and KCR were involved in formal analysis; MET, KH, OVC, RKC, and KCR were involved in visualization; SJA, KCR, and MET were involved in funding acquisition; MET, KH, and KCR were involved in writing – original draft preparation; MET, KH, OVC, DG, RKC, SJA, and KCR were involved in writing – review and editing.

## ETHICS STATEMENT

None required.

## Data Availability

All data generated or analyzed during this study are included in this published article and its corresponding appendices. Additionally, all DNA raw sequencing data files are accessible via the NCBI Sequence Read Archive (SRA) under the accession number PRJNA560077.

## APPENDIX 1

**Table A1:** SNP analysis compared resequenced *S. mutans* UA159 laboratory strain to published *S. mutans* UA159 genome (NC_004350.2)

Reference Position	Type	Length	Reference	Allele	Linkage	Count	Coverage	Frequency	Forward/reverse balance	Average quality	Overlapping annotations	Coding region change	Amino acid change
11066	SNV	1	G	A		857	863	99.3	0.5	36.64	CDS: SMU_13, Gene: SMU_13	NP_720498.1:c.224G > A	NP_720498.1:p.Arg75Gln
385786	SNV	1	C	T		717	722	99.31	0.5	36.11	CDS: brpA, Gene: brpA	NP_720858.1:c.1109C > T	NP_720858.1:p.Ser370Phe
418392	SNV	1	T	C		704	704	100	0.49	36.79	CDS: SMU_448, Gene: SMU_448	NP_720892.1:c.176T > C	NP_720892.1:p.Phe59Ser
552448	Deletion	1	A	–		677	686	98.69	0.49	35.4	CDS: furR, Gene: furR	NP_721026.1:c.393delA	NP_721026.1:p.Asn132fs
808802	SNV	1	C	G		607	607	100	0.5	36.37	CDS: pyrAB, Gene: pyrAB	NP_721270.1:c.1266C > G	
826253	SNV	1	C	G		442	442	100	0.46	36.32	CDS: SMU_875c, Gene: SMU_875c	NP_721284.1:c.214G > C	NP_721284.1:p.Asp72His
842678	SNV	1	G	T		522	523	99.81	0.49	35.9	CDS: galE, Gene: galE	NP_721296.1:c.672G > T	
957858	SNV	1	T	C		152	154	98.7	0.18	36.7	CDS: gtfC, Gene: gtfC	NP_721400.1:c.2121T > C	
957915	SNV	1	C	T		91	91	100	0.01	35.54	CDS: gtfC, Gene: gtfC	NP_721400.1:c.2178C > T	
957924	SNV	1	A	G		85	85	100	0.01	35.74	CDS: gtfC, Gene: gtfC	NP_721400.1:c.2187A > G	
988399	SNV	1	T	G		519	519	100	0.49	36.61			
1148435	SNV	1	G	T		499	500	99.8	0.49	35.58	CDS: fic, Gene: fic	NP_721587.1:c.233C > A	NP_721587.1:p.Ala78Asp
1164137	SNV	1	C	G		571	571	100	0.5	36.2	CDS: pyrF, Gene: pyrF	NP_721601.1:c.144G > C	
1223786	SNV	1	T	C		514	516	99.61	0.48	36.41	CDS: SMU_1297, Gene: SMU_1297	NP_721668.1:c.655T > C	
1237595	SNV	1	G	C		551	551	100	0.5	36.13	CDS: aspB, Gene: aspB	NP_721683.1:c.230C > G	NP_721683.1:p.Ala77Gly
1380458	SNV	1	C	A		605	610	99.18	0.5	36.04	CDS: SMU_1450, Gene: SMU_1450	NP_721803.1:c.162C > A	
1568890	SNV	1	C	T		691	693	99.71	0.5	35.71			
1576987	SNV	1	G	C		716	718	99.72	0.5	34.74	CDS: nrgA, Gene: nrgA	NP_721991.1:c.94C > G	NP_721991.1:p.Arg32Gly
1733172	SNV	1	T	C		56	56	100	0.22	37.38	CDS: aroG, Gene: aroG	NP_722153.1:c.87A > G	
1733175	SNV	1	C	T		35	37	94.59	0.15	37.14	CDS: aroG, Gene: aroG	NP_722153.1:c.84G > A	
1733177	MNV	2	TA	GG		35	37	94.59	0.17	36.3	CDS: aroG, Gene: aroG	NP_722153.1:c.81_82delTAinsCC	NP_722153.1:p.Lys28Gln
1733181	SNV	1	C	T		35	35	100	0.15	37.11	CDS: aroG, Gene: aroG	NP_722153.1:c.78G > A	
1733185	SNV	1	T	G		35	35	100	0.15	37.09	CDS: aroG, Gene: aroG	NP_722153.1:c.74A > C	NP_722153.1:p.Asp25Ala
1733187	SNV	1	C	T		35	35	100	0.15	37.14	CDS: aroG, Gene: aroG	NP_722153.1:c.72G > A	
1733189	SNV	1	G	C		35	35	100	0.15	37.4	CDS: aroG, Gene: aroG	NP_722153.1:c.70C > G	NP_722153.1:p.Gln24Glu
1733194	SNV	1	T	G		35	35	100	0.15	37.74	CDS: aroG, Gene: aroG	NP_722153.1:c.65A > C	NP_722153.1:p.Lys22Thr
1733977	SNV	1	A	T		51	51	100	0.16	37.22	CDS: aroH, Gene: aroH	NP_722154.1:c.315T > A	
1733986	SNV	1	G	A		58	58	100	0.16	37.36	CDS: aroH, Gene: aroH	NP_722154.1:c.306C > T	
1733988	MNV	2	CC	TG		58	58	100	0.16	37.38	CDS: aroH, Gene: aroH	NP_722154.1:c.303_304delGGinsCA	NP_722154.1:p.Met101_Val102delinsIleIle
1733991	MNV	2	TT	GG		58	58	100	0.16	37.34	CDS: aroH, Gene: aroH	NP_722154.1:c.300_301delAAinsCC	NP_722154.1:p.Met101Leu
1733998	SNV	1	G	A		92	92	100	0.23	37.01	CDS: aroH, Gene: aroH	NP_722154.1:c.294C > T	
1734001	SNV	1	G	A		97	97	100	0.24	37.45	CDS: aroH, Gene: aroH	NP_722154.1:c.291C > T	
1734016	SNV	1	G	A		113	113	100	0.26	37.36	CDS: aroH, Gene: aroH	NP_722154.1:c.276C > T	
1892328	Deletion	6	AATAAT	–		632	698	90.54	0.47	35.2	CDS: SMU_2167, Gene: SMU_2167	YP_004853792.1:c.814_819delATTATT	YP_004853792.1:p.Ile272_Ile273del
1932163	Insertion	1	–	T		779	802	97.13	0.49	36.6			

Grey: SNPs common to WT, *cidB* rough, and *cidB* smooth relative to published *S. mutans* UA159 genome (NC_004350.2).

Yellow: SNPs only found in WT (wild‐type laboratory strain UA159 isogenic to both *cidB* mutants).

**Table A2:** SNP analysis compared resequenced *S. mutans cidB* smooth mutant to published S. mutans UA159 genome (NC_004350.2)

Reference Position	Type	Length	Reference	Allele	Linkage	Count	Coverage	Frequency	Forward/reverse balance	Average quality	Overlapping annotations	Coding region change	Amino acid change
9729	SNV	1	A	G		1,039	1,052	98.76	0.49	36.65	CDS: SMU_12, Gene: SMU_12	NP_720497.1:c.98A > G	NP_720497.1:p.Tyr33Cys
11066	SNV	1	G	A		961	962	99.9	0.49	36.16	CDS: SMU_13, Gene: SMU_13	NP_720498.1:c.224G > A	NP_720498.1:p.Arg75Gln
294438	SNV	1	C	T		862	866	99.54	0.49	36.31	CDS: pgi, Gene: pgi	NP_720764.1:c.772C > T	NP_720764.1:p.Arg258Cys
294544	SNV	1	A	G		852	862	98.84	0.5	35.3	CDS: pgi, Gene: pgi	NP_720764.1:c.878A > G	NP_720764.1:p.Glu293Gly
385786	SNV	1	C	T		803	809	99.26	0.49	36	CDS: brpA, Gene: brpA	NP_720858.1:c.1109C > T	NP_720858.1:p.Ser370Phe
418392	SNV	1	T	C		837	838	99.88	0.49	36.8	CDS: SMU_448, Gene: SMU_448	NP_720892.1:c.176T > C	NP_720892.1:p.Phe59Ser
552448	Deletion	1	A	–		738	759	97.23	0.5	34.93	CDS: furR, Gene: furR	NP_721026.1:c.393delA	NP_721026.1:p.Asn132fs
808802	SNV	1	C	G		666	669	99.55	0.47	36.45	CDS: pyrAB, Gene: pyrAB	NP_721270.1:c.1266C > G	
826253	SNV	1	C	G		511	513	99.61	0.45	36.39	CDS: SMU_875c, Gene: SMU_875c	NP_721284.1:c.214G > C	NP_721284.1:p.Asp72His
842678	SNV	1	G	T		637	639	99.69	0.47	35.42	CDS: galE, Gene: galE	NP_721296.1:c.672G > T	
957858	SNV	1	T	C		202	203	99.51	0.18	36.19	CDS: gtfC, Gene: gtfC	NP_721400.1:c.2121T > C	
957915	SNV	1	C	T		148	148	100	0.04	36.69	CDS: gtfC, Gene: gtfC	NP_721400.1:c.2178C > T	
957924	SNV	1	A	G		143	145	98.62	0.05	35.87	CDS: gtfC, Gene: gtfC	NP_721400.1:c.2187A > G	
988399	SNV	1	T	G		619	620	99.84	0.49	36.52			
1164137	SNV	1	C	G		608	608	100	0.5	35.74	CDS: pyrF, Gene: pyrF	NP_721601.1:c.144G > C	
1223786	SNV	1	T	C		605	607	99.67	0.48	36.26	CDS: SMU_1297, Gene: SMU_1297	NP_721668.1:c.655T > C	
1380458	SNV	1	C	A		680	684	99.42	0.49	35.66	CDS: SMU_1450, Gene: SMU_1450	NP_721803.1:c.162C > A	
1568890	SNV	1	C	T		759	764	99.35	0.48	35.17			
1576987	SNV	1	G	C		815	817	99.76	0.5	34.16	CDS: nrgA, Gene: nrgA	NP_721991.1:c.94C > G	NP_721991.1:p.Arg32Gly
1604392	SNV	1	C	G		739	750	98.53	0.49	36.26	CDS: dltA, Gene: dltA	NP_722022.1:c.1396G > C	NP_722022.1:p.Ala466Pro
1733172	SNV	1	T	C		74	74	100	0.15	37.09	CDS: aroG, Gene: aroG	NP_722153.1:c.87A > G	
1733175	SNV	1	C	T		36	36	100	0.08	37	CDS: aroG, Gene: aroG	NP_722153.1:c.84G > A	
1733177	MNV	2	TA	GG		36	36	100	0.1	36.78	CDS: aroG, Gene: aroG	NP_722153.1:c.81_82delTAinsCC	NP_722153.1:p.Lys28Gln
1733181	SNV	1	C	T		36	36	100	0.08	37.47	CDS: aroG, Gene: aroG	NP_722153.1:c.78G > A	
1733185	SNV	1	T	G		36	36	100	0.08	37.61	CDS: aroG, Gene: aroG	NP_722153.1:c.74A > C	NP_722153.1:p.Asp25Ala
1733187	SNV	1	C	T		36	36	100	0.08	37.53	CDS: aroG, Gene: aroG	NP_722153.1:c.72G > A	
1733189	SNV	1	G	C		36	36	100	0.08	36.72	CDS: aroG, Gene: aroG	NP_722153.1:c.70C > G	NP_722153.1:p.Gln24Glu
1733194	SNV	1	T	G		36	36	100	0.08	37.25	CDS: aroG, Gene: aroG	NP_722153.1:c.65A > C	NP_722153.1:p.Lys22Thr
1733977	SNV	1	A	T		53	53	100	0.05	36.89	CDS: aroH, Gene: aroH	NP_722154.1:c.315T > A	
1733986	SNV	1	G	A		60	60	100	0.1	37.43	CDS: aroH, Gene: aroH	NP_722154.1:c.306C > T	
1733988	MNV	2	CC	TG		60	60	100	0.1	37.25	CDS: aroH, Gene: aroH	NP_722154.1:c.303_304delGGinsCA	NP_722154.1:p.Met101_Val102delinsIleIle
1733991	MNV	2	TT	GG		59	61	96.72	0.11	37.3	CDS: aroH, Gene: aroH	NP_722154.1:c.300_301delAAinsCC	NP_722154.1:p.Met101Leu
1733998	SNV	1	G	A		98	98	100	0.21	36.68	CDS: aroH, Gene: aroH	NP_722154.1:c.294C > T	
1734001	SNV	1	G	A		107	108	99.07	0.22	36.59	CDS: aroH, Gene: aroH	NP_722154.1:c.291C > T	
1734016	SNV	1	G	A		141	141	100	0.26	36.82	CDS: aroH, Gene: aroH	NP_722154.1:c.276C > T	
1892328	Deletion	6	AATAAT	–		786	882	89.12	0.47	35.09	CDS: SMU_2167, Gene: SMU_2167	YP_004853792.1:c.814_819delATTATT	YP_004853792.1:p.Ile272_Ile273del
1932163	Insertion	1	–	T		855	888	96.28	0.49	36.44			

Grey: SNPs common to WT, *cidB* rough, and *cidB* smooth relative to published *S. mutans* UA159 genome (NC_004350.2).

Green: SNPs only found in *cidB* smooth variant.

**Table A3:** SNP analysis compared resequenced *S. mutans cidB* rough mutant to published *S. mutans* UA159 genome (NC_004350.2)

Reference Position	Type	Length	Reference	Allele	Linkage	Count	Coverage	Frequency	Forward/reverse balance	Average quality	Overlapping annotations	Coding region change	Amino acid change
9729	SNV	1	A	G		475	477	99.58	0.5	35.62	CDS: SMU_12, Gene: SMU_12	NP_720497.1:c.98A > G	NP_720497.1:p.Tyr33Cys
11066	SNV	1	G	A		454	456	99.56	0.49	35.92	CDS: SMU_13, Gene: SMU_13	NP_720498.1:c.224G > A	NP_720498.1:p.Arg75Gln
294438	SNV	1	C	T		390	392	99.49	0.49	36.57	CDS: pgi, Gene: pgi	NP_720764.1:c.772C > T	NP_720764.1:p.Arg258Cys
294544	SNV	1	A	G		379	384	98.7	0.5	34.81	CDS: pgi, Gene: pgi	NP_720764.1:c.878A > G	NP_720764.1:p.Glu293Gly
385786	SNV	1	C	T		365	369	98.92	0.48	35.79	CDS: brpA, Gene: brpA	NP_720858.1:c.1109C > T	NP_720858.1:p.Ser370Phe
418392	SNV	1	T	C		397	398	99.75	0.49	36.13	CDS: SMU_448, Gene: SMU_448	NP_720892.1:c.176T > C	NP_720892.1:p.Phe59Ser
552448	Deletion	1	A	–		385	393	97.96	0.5	33.84	CDS: furR, Gene: furR	NP_721026.1:c.395del	NP_721026.1:p.Asn132fs
808802	SNV	1	C	G		381	382	99.74	0.49	35.34	CDS: pyrAB, Gene: pyrAB	NP_721270.1:c.1266C > G	
826253	SNV	1	C	G		249	250	99.6	0.46	35.31	CDS: SMU_875c, Gene: SMU_875c	NP_721284.1:c.214G > C	NP_721284.1:p.Asp72His
842678	SNV	1	G	T		355	356	99.72	0.5	34.35	CDS: galE, Gene: galE	NP_721296.1:c.672G > T	
957858	SNV	1	T	C		78	79	98.73	0.17	34.17	CDS: gtfC, Gene: gtfC	NP_721400.1:c.2121T > C	
957915	SNV	1	C	T		52	52	100	0.04	35.83	CDS: gtfC, Gene: gtfC	NP_721400.1:c.2178C > T	
957924	SNV	1	A	G		48	49	97.96	0.06	33.9	CDS: gtfC, Gene: gtfC	NP_721400.1:c.2187A > G	
988399	SNV	1	T	G		345	345	100	0.5	35.32			
1164137	SNV	1	C	G		368	369	99.73	0.49	35.91	CDS: pyrF, Gene: pyrF	NP_721601.1:c.144G > C	
1223786	SNV	1	T	C		312	315	99.05	0.5	35.04	CDS: SMU_1297, Gene: SMU_1297	NP_721668.1:c.655T > C	
1380458	SNV	1	C	A		396	399	99.25	0.49	34.13	CDS: SMU_1450, Gene: SMU_1450	NP_721803.1:c.162C > A	
1568890	SNV	1	C	T		349	350	99.71	0.49	33.59			
1576987	SNV	1	G	C		427	430	99.3	0.5	33.38	CDS: nrgA, Gene: nrgA	NP_721991.1:c.94C > G	NP_721991.1:p.Arg32Gly
1604392	SNV	1	C	G		381	384	99.22	0.49	35.01	CDS: dltA, Gene: dltA	NP_722022.1:c.1396G > C	NP_722022.1:p.Ala466Pro
1733172	SNV	1	T	C		31	32	96.88	0.16	36.77	CDS: aroG, Gene: aroG	NP_722153.1:c.87A > G	
1733175	SNV	1	C	T		15	15	100	0	36.6	CDS: aroG, Gene: aroG	NP_722153.1:c.84G > A	
1733177	MNV	2	TA	GG		14	15	93.33	0.07	36.68	CDS: aroG, Gene: aroG	NP_722153.1:c.81_82delinsCC	NP_722153.1:p.Lys28Gln
1733181	SNV	1	C	T		15	15	100	0	37.87	CDS: aroG, Gene: aroG	NP_722153.1:c.78G > A	
1733185	SNV	1	T	G		15	15	100	0	37.6	CDS: aroG, Gene: aroG	NP_722153.1:c.74A > C	NP_722153.1:p.Asp25Ala
1733187	SNV	1	C	T		15	15	100	0	36.47	CDS: aroG, Gene: aroG	NP_722153.1:c.72G > A	
1733189	SNV	1	G	C		15	15	100	0	36.4	CDS: aroG, Gene: aroG	NP_722153.1:c.70C > G	NP_722153.1:p.Gln24Glu
1733194	SNV	1	T	G		15	15	100	0	35.87	CDS: aroG, Gene: aroG	NP_722153.1:c.65A > C	NP_722153.1:p.Lys22Thr
1733977	SNV	1	A	T		17	17	100	0.26	36.59	CDS: aroH, Gene: aroH	NP_722154.1:c.315T > A	
1733986	SNV	1	G	A		18	18	100	0.25	36.44	CDS: aroH, Gene: aroH	NP_722154.1:c.306C > T	
1733988	MNV	2	CC	TG		18	18	100	0.25	35.36	CDS: aroH, Gene: aroH	NP_722154.1:c.303_304delinsCA	NP_722154.1:p.Met101_Val102delinsIleIle
1733991	MNV	2	TT	GG		17	18	94.44	0.26	36.44	CDS: aroH, Gene: aroH	NP_722154.1:c.300_301delinsCC	NP_722154.1:p.Met101Leu
1733998	SNV	1	G	A		31	31	100	0.31	35.48	CDS: aroH, Gene: aroH	NP_722154.1:c.294C > T	
1734001	SNV	1	G	A		37	37	100	0.31	36.22	CDS: aroH, Gene: aroH	NP_722154.1:c.291C > T	
1734016	SNV	1	G	A		47	47	100	0.32	34.11	CDS: aroH, Gene: aroH	NP_722154.1:c.276C > T	
1932163	Insertion	1	–	T		440	458	96.07	0.49	36.09			

Grey SNPs common to WT, *cidB* rough, and *cidB* smooth relative to published *S. mutans* UA159 genome (NC_004350.2).

Green: SNPs only found in *cidB* rough variant.
